# Influenza-associated mortality in hospital care: a retrospective cohort study of risk factors and impact of oseltamivir in an English teaching hospital, 2016 to 2017

**DOI:** 10.2807/1560-7917.ES.2019.24.44.1900087

**Published:** 2019-10-31

**Authors:** Mark Reacher, Ben Warne, Lucy Reeve, Neville Q. Verlander, Nicholas K. Jones, Kyriaki Ranellou, Silvana Christou, Callum Wright, Saher Choudhry, Maria Zambon, Clare Sander, Hongyi Zhang, Hamid Jalal

**Affiliations:** 1 Public Health England Field Service, Cambridge Institute of Public Health, Cambridge, United Kingdom; 2 Public Health England and Cambridge Universities Hospitals NHS Foundation Trust Cambridge, Cambridge, United Kingdom; 3 Cambridge University Hospitals NHS Foundation Trust, Cambridge, United Kingdom; 4 Statistics Unit, Statistics, Modelling and Economics Department, National Infection Service – Data and Analytical Sciences, Public Health England, London, United Kingdom; 5 Division of Virology, Department of Pathology, University of Cambridge, United Kingdom; 6 National Infection Service, Public Health England, London, United Kingdom

**Keywords:** Historic Cohort study, influenza, in-patient mortality, risk factors, logistic regression, multivariable logistic regression

## Abstract

**Background:**

Evidence of an oseltamivir treatment effect on influenza A(H3N2) virus infections in hospitalised patients is incomplete.

**Aims:**

This cohort study aimed to evaluate risk factors for death among PCR-confirmed hospitalised cases of seasonal influenza A(H3N2) of all ages and the impact of oseltamivir.

**Methods:**

Participants included all 332 PCR-confirmed influenza A(H3N2) cases diagnosed between 30 August 2016 and 17 March 2017 in an English university teaching Hospital. Oseltamivir treatment effect on odds of inpatient death was assessed by backward stepwise multivariable logistic regression analysis.

**Results:**

The odds of death were reduced by two thirds (odds ratio (OR): 0.32; 95% confidence interval (CI): 0.11–0.93), in inpatients treated with a standard course of oseltamivir 75 mg two times daily for 5 days – compared with those untreated with oseltamivir, after adjustment for age, sex, current excess alcohol intake, receipt of 2016/17 seasonal influenza vaccine, serum haemoglobin and hospital vs community attribution of acquisition of influenza.

**Conclusions:**

Oseltamivir treatment given according to National Institutes of Clinical Excellence (NICE); United States Centres for Disease Control and Prevention (CDC); Infectious Diseases Society of America (IDSA) and World Health Organization (WHO) guidelines was shown to be effective in reducing the odds of mortality in inpatients with PCR-confirmed seasonal influenza A(H3N2) after adjustment in a busy routine English hospital setting. Our results highlight the importance of hospitals complying with relevant guidelines for prompt seasonal influenza PCR testing and ensuring standard oseltamivir treatment to all PCR-confirmed cases of seasonal influenza.

## Introduction

Seasonal influenza, which recurs annually in winter, remains a leading cause of morbidity and mortality worldwide and a major challenge to hospitals, which face severe pressures during this period of the year [1,2]. Hospitalisation and mortality from influenza disproportionately affect the elderly [3-5], with the risk of mortality increasing with age over 65 years [6,7]. Other factors associated with mortality include underlying comorbidities [5,8], delay in presentation to medical care [8] and viral subtype, with infection with influenza A(H3N2) virus strains being of highest risk [3,5,7].

Circulating influenza virus strains and vaccine effectiveness vary from season to season [9-11]. Understanding risk factors for poor outcome in seasonal influenza is therefore important in optimising the prevention and management of high-risk patients presenting to primary and secondary care and requires appropriately designed observational studies [9,12].

In common with many hospitals in the northern hemisphere winter of 2016/17, Cambridge University Hospitals (CUH) National Health Service (NHS) Foundation Trust was severely challenged by large numbers of patients admitted with influenza virus infection [13].

During this season, influenza A(H3N2) virus strain predominated and seasonal influenza vaccine was estimated to have moderate effectiveness of between 30% and 40% with lower levels of effectiveness in older patients [14-17].

Although recommended by several national and international agencies for the treatment of seasonal influenza in patients requiring hospitalisation, including the National Institute for Health and Care Excellence (NICE) [18,19], the United States (US) Centers for Disease Control and Prevention (CDC) [20,21], the Infectious Disease Society of America (IDSA) [22] and the World Health Organization (WHO) [16], the evidence of an effect of oseltamivir on influenza-associated mortality in hospitalised patients is limited [10]. Randomised controlled trials (RCTs) have predominantly been undertaken in outpatients and have indicated that oseltamivir reduced symptom duration and lower respiratory tract complications in otherwise healthy adults [23,24].

A retrospective cohort study was undertaken in our hospital of the clinical management of inpatients diagnosed by PCR with influenza A(H3N2) virus strain infection during the 2016/17 season to better understand risk factors for inpatient death and whether any effect of a standard course of oseltamivir 75 mg twice daily for 5 days was observed.

## Methods

### Institutional setting and approval

The CUH NHS Foundation Trust is a 1,100-bed university teaching hospital providing district general hospital services for Cambridgeshire and surrounding counties and specialised tertiary-level services across the East of England with a population of approximately six million.

### Ethical statement

The study was registered with the Safety and Quality Support department of CUH NHS Foundation Trust under a Service Evaluation/Outbreak Investigation remit (Project Registration Number PRN 7053; 15 January 2018).

### Legal basis for processing personal identifying information

Processing and analysis of individual patient level data was undertaken in compliance with the General Data Protection Regulations [25,26].

### Virological and bacteriological methods

A nose and a throat swab placed in a single tube of viral transport medium were collected from each patient. Detection of respiratory viruses was done by using a multiplex real-time PCR assay as described previously [27,28]. Bacteriology specimens were processed using standard Public Health England (PHE) methods.

### Individuals considered in the study

Individuals in this study were patients of any age admitted to our hospital with a laboratory-confirmed diagnosis of influenza A(H3N2) virus strain infection, between 20 August 2016 and 17 March 2017. They were identified through our hospital laboratory information system (LIMS) and linked to electronic hospital medical record and general practitioner (GP) information systems. Death or discharge alive was recorded for the admission episode during which the virological swab testing positive for the influenza A(H3N2) virus strain was obtained.

### Clinical data collection

Demographic and clinical details of the admission episode, date and time of admission, discharge, transfer and death, full blood count, blood chemistry, C-reactive protein, virology and bacteriology test results, oseltamivir prescriptions and receipt of seasonal influenza vaccination were extracted from the electronic hospital medical record and GP information systems described earlier to a structured epidemiological proforma.

Active medical conditions were recorded and the non-age adjusted Charlson comorbidity index score calculated [29]. Risk factors and conditions not included in the Charlson index were also recorded, comprising radiological evidence of pneumonia, current smoker, excessive alcohol use, admission with trauma, admission for surgery, pregnancy, hypertension, body mass index (BMI) ≥ 40 kg/m^2^ and receipt of respiratory support. Respiratory support was categorised as oxygen therapy, continuous positive airways pressure (CPAP), non-invasive ventilation (NIV) and invasive ventilation. Immune suppression was categorised as absent or present and, if present, categorised as: high dose steroids ≥ 40 mg of prednisolone daily or equivalent; receiving or within 6 months of receiving chemotherapy or generalised radiotherapy, organ transplant or bone marrow transplant.

The clinical data abstraction proforma for oseltamivir exposure posed the question ‘was this patient prescribed oseltamivir, yes or no? If yes, did this patient receive standard oseltamivir treatment 75 mg twice daily for 5 days, yes or no? If no, describe why not.’

The British National Formulary (BNF) standard course of oseltamivir for treatment of seasonal influenza is 75 mg twice daily for 5 days. For children modified doses are given according to body weight. Patients whose treatment was completed according to this standard were categorised as standard course.

In renal failure the standard 5-day dose is reduced in adults to 30 mg twice daily if estimated glomerular filtration rate (eGFR) is 30–60 mL/minute/1.73 m^2^ or to 30 mg once daily if eGFR is 10–30 mL/minute/1.73 m^2^. In children this is 40% of normal dose for weight twice daily if eGFR is 30–60 mL/minute/1.73 m^2^ or 40% of normal dose by weight once daily if eGFR is 10–30 mL/minute/1.73 m^2^. In patients with immune suppression the standard course is extended from 5 to 10 days. Patients whose treatment was modified for renal failure or immune suppression in accordance with the BNF were categorised as having received an appropriately modified standard course.

Cases whose oseltamivir treatment was neither standard, 75 mg twice daily for 5 days, nor appropriately modified, were categorised as having received a non-standard course of oseltamivir.

Oseltamivir exposure was categorised from these responses as not received (category 1); received a non-standard course (category 2); received an appropriately modified course (category 3); or received a standard course 75 mg twice daily for 5 days (category 4) compared with the BNF [30]. Time of starting oseltamivir was calculated from the date of onset of influenza symptoms to the date of the first dose given in the pharmacy record, which is part of the electronic patient record.

Alcohol exposure was obtained from a table entitled ‘active conditions on admission’ which sought a response for the item ‘Excessive alcohol use’ yes or no.

Hospital and primary care records were reviewed for evidence of having received an NHS approved seasonal influenza vaccine for the 2016/17 influenza season. The clinical data abstraction questionnaire field read ‘Record of receipt of an NHS approved 16/17 seasonal influenza vaccine? yes or no.’

The place of acquisition of influenza A(H3N2) virus strain infection was attributed to the community when symptom onset was before admission or within 2 days of admission and if no outpatient contact with the hospital had occurred within 2 days of onset of symptoms. Hospital acquisition was attributed where onset of symptoms was more than 2 days after admission or if outpatient, dialysis or chemotherapy day centre contact at our hospital had occurred within 2 days of onset of influenza illness.

### Statistical analysis

Statistical analysis was undertaken in STATA15.1. using single variable and multivariable regression methods [31]. Single variable analysis associations between inpatient death and individual risk factors, including the individual components of the Charlson comorbidity index and the non-age adjusted Charlson comorbidity index, were examined by logistic regression using likelihood ratio tests (LRT) and p values and odds ratios (OR) and 95% confidence intervals (CI) determined. Variables with p < 0.2 by LRT in the single variable analysis and sex were considered in the stepwise multivariable logistic regression analysis. Appropriate functional forms for continuous variables were determined by successively fitting cubic, quadratic and linear functions and selecting the simplest for which no improvement in fit was observed by LRT in both single and multivariable analyses.

The initial model consisted of sex and variables with p ≤ 0.01 by LRT from the single variable analysis. A backwards stepwise procedure was undertaken dropping non-confounding variables with the largest LRT p > 0.1 one at a time. A variable was judged to be confounding if its omission resulted in a change of more than 20% in the OR in one or more of the parameters still in the model. The process continued until no further variables could be removed.

Variables with p ≤ 0.1 in the single variable analysis were then added in a forward procedure and the above procedure repeated, at the end of which the remaining variables with p > 0.1 were added and the backwards stepwise procedure implemented.

The variables for the category of oseltamivir treatment completed and receipt of 2016/17 seasonal influenza vaccine were then added to the model. Following this, variables which had been dropped from the model were again added singly and then removed before adding another to check that they should still be omitted. Variables found to be substantial confounders were then added to the model. The appropriate functions of the continuous variables still in the model were again determined to give the final multivariable model of risk factors independently associated with inpatient death, adjusting for the category of oseltamivir treatment received and receipt of an NHS approved 2016/17 seasonal influenza vaccine.

A second multivariable model was fitted replacing the variable for the category of oseltamivir treatment with the variable for the delay between the date of onset of influenza illness and the date of commencing oseltamivir treatment.

### Interactions

Interaction terms were tested between the categories of oseltamivir treatment received, delay in receipt of oseltamivir, receipt of approved 2016/17 seasonal influenza vaccine and if immune suppressed. The variable of the delay was considered in two two-way interactions, one being with the receipt of seasonal vaccine and the other immune suppressed. The two-way interaction between immune suppression and seasonal vaccine receipt and the three-way interaction between delay, seasonal vaccine receipt and immune suppression were tested. These interactions were considered in both the single and multivariable analysis.

## Results

### Single variable analysis

The cohort comprised 332 patients with a mean age of 68.6 years (range: 0–102) of whom 175 (52.7%) were female. A total of 32 (9.6%) of these patients died during admission (Table 1). The time from admission to death, discharge or transfer to other hospitals was median 7 days (interquartile range: 3–23). Eight (2.4%) patients were aged under 18 years: one under 1 year; three aged 2 years; one aged 5 years; one aged 8 years; and two aged 17 years, none of whom expired during admission. Twenty-six (8.1%) of the patients were admitted to an intensive care unit (ICU).

**Table 1:** Analysis of single variables potentially associated with influenza A(H3N2) virus strain infection related deaths, Cambridge, England, 2016/17 (n = 332)

Variable	Category or measure	Expired	Not expired	OR (95% CI)	p value
Age at positive specimen (years)	Minimum	34	0	1.04 (1.01–1.06) per year	< 0.001
	25th centile	71	53		
	Median	84	74		
	75th centile	88	85		
	Maximum	102	101		
Sex	Female	13	162	Reference	0.15
	Male	19	138	1.72 (0.82–3.60)	
Body mass index (BMI) ≥ 40 Kg/m^2^	Yes	1	5	Reference	0.6
	No	31	295	1.90 (0.22–16.8)	
Pregnancy	Pregnant	0	3	0.00 (not estimable)	0.3
	Not pregnant	13	159	Reference	
	Male	19	138	1.68 (0.80–3.54)	
Oseltamivir course completed	1. Not given	7	66	Reference	0.7
	2. Non-standard course	3	18	1.57 (0.37–6.70)	
	3. Appropriately modified	6	40	1.41 (0.44–4.51)	
	4. Standard course	16	176	0.86 (0.34–2.18)	
Days between onset of influenza illness and first dose of oseltamivir	Minimum	0	0	0.89 (0.74–1.06) per day delay	0.4
	25th centile	2	2		
	Median	3	3		
	75th centile	5	5		
	Maximum	11	18		
Current smoker	Yes	2	29	0.60 (0.13–2.68)	0.5
	No	23	200	Reference	
Long-term oxygen therapy	Yes	1	2	4.69 (0.41–53.6)	0.3
	No	24	225	Reference	
Hypertension	Yes	14	111	1.32 (0.63–2.77)	0.5
	No	18	189	Reference	
Trauma	Yes	1	4	2.39 (0.26–22.0)	0.5
	No	31	296	Reference	
Excessive alcohol use	Yes	3	5	6.10 (1.39–26.8)	0.03
	No	29	295	Reference	
Surgery	Yes	3	13	2.28 (0.61–8.48)	0.25
	No	29	287	Reference	
Immune suppressed	Yes	3	61	0.40 (0.12–1.37)	0.11
	No	29	238	Reference	
Receipt of 2016/17 influenza vaccine	Yes	11	110	0.90 (0.42–1.95)	0.8
	No	21	190	Reference	
Steroids	Yes	0	2	0.00 (not estimable)	0.5
	No	32	298	Reference	
Chemotherapy	Yes	0	21	0.00 (not estimable)	0.04
	No	32	279	Reference	
Radiotherapy	Yes	1	1	9.65 (0.59–158)	0.14
	No	31	299	Reference	
Organ transplant	Yes	0	12	0.00 (not estimable)	0.12
	No	32	288	Reference	
Bone marrow transplant	Yes	0	4	0.00 (not estimable)	0.4
	No	32	296	Reference	
Immune deficiency syndrome	Yes	0	0	Not estimable	NA
	No	32	300	Reference	
Other category of immune suppression	Yes	2	21	0.89 (0.20–3.96)	0.9
	No	30	279	Reference	
Haemoglobin concentration g/L	Minimum	68	45	0.98 (0.96–0.99) per g/L	0.01
	25th centile	98.5	108		
	Median	112	123		
	75th centile	124	135		
	Maximum	153	175		
Total white cell count × 10^9^/L	Minimum	2.1	0	QF^a^	0.008
	25th centile	7.7	5.3		
	Median	10.1	7.3		
	75th centile	14.3	9.6		
	Maximum	48.5	53.6		
Lymphocyte count × 10^9^/L	Minimum	0.2	0	0.88 (0.57–1.37) per count per × 10^9^/L	0.4
	25th centile	0.5	0.5		
	Median	0.8	0.8		
	75th centile	1.2	1.2		
	Maximum	2.3	46.7		
C-reactive protein mg/L	Minimum	5	1	1.00 (1.00–1.01)	0.01
	25th centile	26.3	19.6		
	Median	64.9	39.9		
	75th centile	164	76.6		
	Maximum	431	479		
Creatinine mmol/L	Minimum	48	8	QF^b^	0.002
	25th centile	69	64		
	Median	99	84		
	75th centile	114	111		
	Maximum	187	742		
Urea mmol/L	Minimum	4.5	1.6	QF^c^	< 0.001
	25th centile	7.2	4.6		
	Median	9.2	6.2		
	75th centile	11	8.6		
	Maximum	18.6	39.2		
Glucose mmol/L	Minimum	5.1	3.4	1.04 (0.95–1.13) per mmol/L	0.4
	25th centile	6.7	6		
	Median	7.8	6.9		
	75th centile	8.9	8.7		
	Maximum	24	36.2		
Required oxygen	Yes	8	32	2.79 (1.16–6.73)	0.03
	No	24	268	Reference	
Continuous positive airways pressure	Yes	1	3	3.20 (0.32–31.6)	0.4
	No	31	297	Reference	
Non-invasive ventilation	Yes	3	3	10.2 (1.98–53.1)	0.01
	No	29	297	Reference	
Invasive ventilation	Yes	5	22	2.34 (0.82–6.68)	0.14
	No	27	278	Reference	
Charlson comorbidity index	Minimum	0	0	1.21 (1.03–1.43)	0.03
	25th centile	2	1		
	Median	2	2		
	75th centile	4	3		
	Maximum	8	10		
Cycle threshold (CT) value	Minimum	19	16	0.96 (0.88–1.04)	0.3
	25th centile	23	27		
	Median	27	30		
	75th centile	32	32		
	Maximum	38	41		
Place of acquisition of infection	Community	15	219	Reference	0.003
	Hospital	17	81	3.06 (1.46–6.42)	
Admitted from	Own home	25	251	Reference	0.5
	Residential care	4	28	1.43 (0.47–4.42)	
	Another hospital	3	13	2.32 (0.62–8.68)	
	Other	0	4	0.00 (not estimable)	
Oseltamivir prescribed for post-exposure prophylaxis	Yes	2	8	2.43 (0.49–12.0)	0.3
	No	30	292	Reference	
Pneumonia	No imaging	2	34	Reference	0.005
	No pneumonia on image	16	214	1.27 (0.28–5.78)	
	Pneumonia on image	14	52	4.58 (0.98–21.4)	
Myocardial infarction	Yes	6	32	1.93 (0.74–5.05)	0.2
	No	26	268	Reference	
Congestive heart failure	Yes	5	33	1.50 (0.54–4.16)	0.5
	No	27	267	Reference	
Peripheral vascular disease	Yes	3	8	3.78 (0.95–15.0)	0.09
	No	29	292	Reference	
Cerebro vascular disease	Yes	8	47	1.79 (0.76–4.23)	0.2
	No	24	253	Reference	
Dementia	Yes	8	38	2.30 (0.96–5.48)	0.08
	No	24	262	Reference	
Chronic lung disease	Yes	16	88	2.41 (1.15–5.03)	0.02
	No	16	212	Reference	
Connective tissue disease	Yes	2	21	0.89 (0.20–3.96)	0.9
	No	30	279	Reference	
Peptic ulcer	Yes	3	6	5.07 (1.20–21.3)	0.047
	No	29	294	Reference	
Mild liver disease	Yes	1	2	4.81 (0.42–54.5)	0.3
	No	31	298	Reference	
Moderate or severe liver disease	Yes	3	9	3.34 (0.86–13.0)	0.11
	No	29	291	Reference	
Diabetes without end organ damage	Yes	5	49	0.95 (0.35–2.58)	0.9
	No	27	251	Reference	
Diabetes with end organ damage	Yes	2	15	1.27 (0.28–5.81)	0.8
	No	30	285	Reference	
Hemiplegia	Yes	1	13	0.71 (0.09–5.63)	0.7
	No	31	287	Reference	
Moderate or severe kidney disease	Yes	5	46	1.02 (0.37–2.79)	0.97
	No	27	254	Reference	
Tumour without metastasis	Yes	3	22	1.31 (0.37–4.63)	0.7
	No	29	278	Reference	
Tumour with metastasis	Yes	1	6	1.58 (0.18–13.6)	0.7
	No	31	294	Reference	
Leukaemia	Yes	0	11	0.00 (not estimable)	0.13
	No	32	289	Reference	
Lymphoma	Yes	0	16	0.00 (not estimable)	0.07
	No	32	284	Reference	

OR: odds ratio; CI: confidence interval; QF: quadratic function; NA: not applicable.

^a^ QF: linear part OR: 1.28 (95% CI: 1.11–1.47); quadratic part OR: 1.00 (95% CI: 0.99–1.00).

^b^ QF: linear part OR: 1.05 (95% CI: 1.01–1.09); quadratic part OR: 1.00 (95% CI: 1.00–1.00).

^c^ QF: linear part OR: 2.45 (95% CI: 1.48–4.06); quadratic part OR: 0.97 (95% CI: 0.94–0.99).

Oseltamivir was not prescribed for 73 of the 332 (22%) of patients. Among these patients, the reasons for not having been prescribed oseltamivir were because more than 48 hours had elapsed since onset of symptoms (n = 10; 13.7%), discharged before the positive PCR test result for influenza A(H3N2) virus strain was available (n = 18; 24.7%), clinically improved (n = 8; 11%), died before the test result was received (n = 2; 2.7%), drugs stopped for palliative care (n = 1; 1.4%), and no reason given (n = 34; 46.6%). No patients were detected as having received oseltamivir before admission.

Significantly raised odds (p < 0.05) for inpatient death were observed for age (OR: 1.04; 95% CI: 1.01–1.06) per year, Charlson comorbidity index (OR: 1.21; 95% CI: 1.03–1.43) per unit, excessive alcohol use (OR: 6.10; 95% CI: 1.39–26.8), peptic ulcer (OR: 5.07; 95% CI: 1.2–21.3), radiological evidence of pneumonia (OR: 4.58; 95% CI: 0.98–21.4), requirement for oxygen therapy (OR: 2.79; 95% CI: 1.16–6.73) and receipt of non-invasive ventilation (OR: 10.2; 95% CI: 1.98–53.1). Acquisition of infection within hospital compared with community was also associated with raised odds (OR: 3.06; 95% CI: 1.46–6.42).

Completion of a standard course of oseltamivir 75 mg two times daily for 5 days compared with no treatment with oseltamivir showed a non-significant protective effect (OR: 0.86; 95% CI: 0.34–2.18). No protection was observed for non-standard courses of oseltamivir (OR: 1.57; 95% CI: 0.37–6.70) or appropriately modified course consistent with the BNF (OR: 1.41; 95% CI: 0.44–4.51). No significant association was seen for delay starting oseltamivir (OR: 0.89; 95% CI: 0.74–1.06), with having received the 2016/17 seasonal influenza vaccine (OR: 0.90; 95% CI: 0.42–1.95) or with being immune suppressed (OR: 0.40; 95% CI: 0.12–37).

The significance of interaction terms were delay in starting oseltamivir and 2016/17 seasonal influenza vaccine p = 0.14, delay and immune suppression p = 0.7, immune suppression and seasonal influenza vaccine p = 0.2, and delay in starting oseltamivir and immune supressed and seasonal influenza vaccine p > 0.999.

Six patients, all of whom survived, were confirmed to have bacterial infection synergistic with influenza A(H3N2) virus strain infection by positive cultures for *Streptococcus pneumoniae* or *Haemophilus influenzae* in blood or sputum. None of the patients who expired had evidence of synergistic bacterial infection with influenza A(H3N2) virus strain infection.

### Multivariable analysis

The variables age, sex, place of acquisition, serum haemoglobin concentration, excessive alcohol use, category of oseltamivir course completed and receipt of 2016/17 seasonal influenza vaccine met the criteria for inclusion in the final multivariable model (Table 2).

**Table 2:** Final multivariable analysis of variables independently associated with inpatient death, Cambridge, England, 2016/17 (n = 329)^a^

Variable	Category	OR (95% CI)	p value
Age	NA	1.06 per year (1.03–1.10)	< 0.001
Sex	Female	Reference	0.19
	Male	1.73 (0.76–1.94)	
Place of acquisition	Community	Reference	0.16
	Hospital	1.82 (0.79–4.23)	
Haemoglobin concentration g/L	NA	0.97 (0.95–1.00) per g/L	0.02
Excessive alcohol use	Yes	13.2 (1.93–90.5)	0.01
	No	Reference	
Oseltamivir course completed	1.Not given	Reference	0.23
	2. Non-standard course	0.3 (0.06–1.54)	
	3. Appropriately modified course	0.34 (0.09–1.28)	
	4. Standard course	0.32 (0.11–0.93)	
Receipt of 2016/17 seasonal influenza vaccine	Yes	0.63 (0.26–1.49)	0.3
	No	Reference	

CI: confidence interval; NA: not applicable; OR: odds ratio.

^a ^Of 332 patients, 329 had serum haemoglobin concentration results.

Cases who received a BNF standard course of oseltamivir of 75 mg two times daily for 5 days were significantly protected against death compared with those not given oseltamivir (OR: 0.32; 95% CI: 0.11–0.93) A protective though non-significant association was also observed for receipt of a non-standard course of oseltamivir (OR: 0.3; 95% CI: 0.06–1.54) and a BNF appropriately modified course of oseltamivir (OR: 0.34; 95% CI: 0.09–1.28). Receipt of the 2016/17 seasonal influenza vaccine was not significantly protective (OR: 0.63; 95% CI: 0.29–1.49).

History of excessive alcohol use was associated with a 13-fold (OR: 13.2; 95% CI: 1.93–90.5) higher odds of death. Higher serum haemoglobin was significantly protective (OR:  0.97; 95% CI: 0.95–1.00) per gram per litre. Males and those whose acquisition of infection was in hospital had non-significantly raised odds (OR: 1.73; 95% CI: 0.76–1.94 and OR: 1.82; 95% CI: 0.79–4.23 respectively).

In a second multivariable model, the category of oseltamivir course received was replaced by the delay in days between onset date and starting date of oseltamivir treatment (Table 3). This delay was not significantly associated with inpatient death (OR: 0.92; 95% CI: 0.77–1.09). The remaining variables had similar odds as in the first multivariable model Tables 2 and 3.

**Table 3:** Multivariable analysis with delay in days between symptom onset and start of oseltamivir treatment, Cambridge, England, 2016/17 (n = 299)^a^

Variable	Category	OR (95% CI)	p value
Age	NA	1.06 per year (1.03–1.10)	< 0.001
Sex	Female	Reference	0.19
	Male	1.74 (0.75–4.01)	
Place of acquisition	Community	Reference	0.4
	Hospital	1.43 (0.57–3.58)	
Haemoglobin concentration g/L	NA	0.97 (0.95–0.99) per g/L	0.01
Excessive alcohol use	Yes	16.7 (1.77–156)	0.02
	No	Reference	
Time delay from onset of symptoms to starting oseltamivir in days	NA	0.92 per day (0.77–1.09)	0.1
Receipt of 2016/17 seasonal influenza vaccine	Yes	0.59 (0.25–1.40)	0.22
	No	Reference	

CI: confidence interval; NA: not applicable; OR: odds ratio.

^a ^Of 332 patients, 299 had information on time delay and haemoglobin concentration.

None of the following interactions tested in the model shown in Table 3 reached statistical significance: delay in starting oseltamivir and receipt of 2016/17 seasonal influenza vaccine p = 0.6, delay and immune suppressed p = 0.6, immune suppressed and receipt of 2016/17 seasonal influenza vaccine p = 0.3, and delay and immune suppressed and receipt of 2016/17 seasonal influenza vaccine p > 0.999.

Variables significantly associated with raised odds in single variable analysis p< 0.05 (Table 1) but excluded in our multivariable model building (Tables 2 and 3) were chemotherapy, total white cell count, serum C-reactive protein, serum creatinine, serum urea, supplementary oxygen therapy, non-invasive ventilation, Charlson comorbidity index, radiological evidence of pneumonia, chronic lung disease, and peptic ulcer.

## Discussion

This study of a large cohort of laboratory-confirmed seasonal influenza A (H3N2) cases admitted to a United Kingdom (UK) NHS hospital measured the odds of all-cause inpatient mortality. Our study has chronicled the effectiveness of oseltamivir in reducing inpatient mortality in a hospital typical of the UK NHS in winter season 2016/17 for the influenza A(H3N2) virus strain and that a proportion of patients had not received oseltamivir treatment at standard BNF dose according to NICE and WHO guidelines for several operational reasons, some of which were amenable to improvement.

While RCTs of oseltamivir treatment of influenza virus infection have been conducted in outpatients, there are currently no completed placebo-controlled RCTs of oseltamivir for treatment of influenza in hospitalised patients, thus the need for observational studies [10].

The need for observational studies to supplement RCTs is recognised [9] and can provide up to date measures of effectiveness of interventions and treatments in routine clinical settings with external validity. This is important in seasonal influenza where the effectiveness of vaccines and the virulence of circulating influenza virus strains vary over time [10,11].

We identified clinical parameters in single variable analysis recognised as risk factors for poor outcome including elevated total white cell count and C-reactive protein, pneumonia, and the need for supplementary oxygen and ventilation [32], but these did not meet our inclusion criteria for our final multivariable models.

The variables meeting our selection criteria for our final multivariable model (Table 2) were age, sex, place of acquisition, serum haemoglobin concentration, excessive alcohol use, category of oseltamivir treatment and receipt of 2016/17 seasonal influenza vaccine.

The significant independent protective association against inpatient death of higher serum haemoglobin concentration is consistent with this being a marker of good general nutrition and better health [33]. Excessive alcohol use emerged as a major confounder and may be explained at least in part, by its association with severe liver disease, which is a well-recognised risk factor for severe influenza virus infection. We combined moderate and severe liver disease as a single variable as required for the Charlson comorbidity index but this was not associated with raised odds of inpatient death in our single variable analysis (Table 1). 

Recorded receipt of 2016/17 seasonal influenza vaccine neither protected our cases from infection with influenza (A(H3N2) virus strain nor modified the protective effect of oseltamivir on inpatient death as shown by the non-significance of its interaction terms. These results are in keeping with the observed poor effectiveness of the vaccine against influenza A(H3N2) particularly in the elderly [14,15,17].

The effectiveness of oseltamivir in the prevention and treatment of seasonal and pandemic influenza remains a subject of debate [34-36].

Acceptance of the protective effect of oseltamivir against inpatient mortality in seasonal influenza A(H3N2) virus strain infection shown in our study, may be perceived to be at variance with meta-analyses of RCTs, which have concluded that the benefits of oseltamivir treatment are confined to shortening of influenza symptoms [23,24,37,38]. However, these trials have been done in outpatients and almost exclusively in younger adults with no comorbidities and are probably not generalisable to the largely elderly hospitalised population in this study [9,39] for whom oseltamivir treatment is recommended by NICE, IDSA, CDC and WHO [16,18,19,22,40]. Our findings concur with meta-analyses of observational studies concluding a protective effect of oseltamivir on mortality in patients hospitalised with Influenza [41,42]. However, Jones and Wolkewitz have raised concerns about the meta-analysis by Muthuri et al. on handling of time-dependent treatment and the absence of follow up beyond hospital discharge [43,44].

In keeping with previous observational studies [42,45], we found no evidence to support the notion that the protective benefit of oseltamivir was limited in this high-risk population to within 48 hours after the onset of symptoms. The additional benefit found in our cohort may reflect the higher proportions of elderly and immunocompromised patients who have longer times from symptom onset to cessation of viral replication [46,47].

The failure to give oseltamivir treatment to cases of influenza A (H3N2) virus strain admitted to hospital may expose cases to an avoidable threefold higher odds of inpatient mortality than would have been the case had they been treated and as recommended by national and international guidance. Infection ascribed to being acquired in hospital as compared to being acquired in the community met our selection criteria for inclusion in our final multivariable models and weak but non-significant association with raised odds of inpatient mortality was seen. This may be explained by greater comorbidity in the former group of patients.

We measured the odds of inpatient mortality as our outcome using logistic regression and showed a protective effect of oseltamivir treatment in hospitalised patients. Our conclusion of a protective effect of oseltamivir against inpatient mortality in hospitalised patients is congruent with the conclusion of a recent systematic review of systematic reviews [36].

Our conclusions contrast with studies measuring duration of admission as well as inpatient mortality using survival analysis. A case for modelling discharge from the population at risk as a competing hazard for death during admission has been made [44,48]. Multistate models were used comprising hospital admission, oseltamivir treatment, discharge, and death timed from onset of influenza symptoms using 1,391 case records of confirmed pandemic influenza A(H1N1) virus strain infection collected during 2009 and 2010 in the UK by the UK Flu collaboration [48,49]. A corrected hazard ratio (HR) of death in hospital associated with oseltamivir treatment was found to be non-significant (HR: 1.03; 95% CI: 0.64–1.66) but significant for discharge (HR: 1.89; 95% CI: 1.65–2.16). Lytras et al. modelled discharge from ICUs alive as a competing hazard for death within the ICUs, for oseltamivir exposure dichotomised to early (≤ 48 hours after onset) vs later (> 48 hours) for influenza A(H3N2) virus strain infection in a cohort of 1,330 patients admitted to ICUs in Greece over eight influenza seasons between 2010 and 2018 [50]. Although early treatment with oseltamivir was associated with significantly lower mortality (relative risk (RR): 0.69; 95% CI:  0.49–0.94), the authors ascribed this effect purely to increased cause-specific hazard for discharge.

The absence of demonstrable protective effect of oseltamivir on inpatient mortality in these studies contrast to our results. This could be explained by the differences in the influenza A virus strains being studied; treating discharge from the population at risk as a competing hazard with patient death rather than considering death alone as the outcome; and differences in adjustment for confounding. For example, no adjustment was made for excessive alcohol use in these studies, while we observed excessive alcohol use to be a significant confounder of the relationship between seasonal influenza A(H3N2) virus strain infection and inpatient death.

### Limitations

Our study was limited by being for a single season from a single hospital and for cases only infected by influenza A(H3N2) virus strain. We enrolled all influenza A(H3N2) virus strain positive patients to our study cohort, but it is possible that some cases may still have been missed from viral swabbing. The small proportion of children we recruited may reflect this. Because we had few children aged under 18 years, we were unable to draw specific conclusions on this age group. It is possible that we may not have recorded fully oseltamivir given before admission to hospital, but we think this is unlikely because data abstractors were clinicians reviewing the entire clinical record including drugs given before admission and primary care physician referral letters and with a study remit to search for reasons why a completed standard course of oseltamivir had not been given. No case of oseltamivir having been given before admission was reported.

It is possible that a further proportion of our patients had received seasonal influenza vaccine than we recorded, because influenza vaccination is offered in pharmacies and supermarkets from which vaccination records may not have been completely transferred to the primary care records which we used. We do not think this would have led to a major impact on our conclusions because we did not detect a protective effect for seasonal influenza vaccination alone or as an interaction term with oseltamivir, and because the 2016/17 influenza vaccine was of low effectiveness particularly in the elderly [14]. Corroboration of our results in future seasons in other centres and for other virus strains of seasonal influenza is desirable.

## Conclusions

Standard oseltamivir treatment of 75 mg twice daily for 5 days was shown to be effective in reducing the odds of inpatient mortality by two thirds (OR: 0.32; 95% CI: 0.11–0.93) in patients hospitalised with PCR-confirmed seasonal influenza A(H3N2) virus strain infection in a routine NHS setting.

Rapid treatment with oseltamivir requires rapid diagnosis of seasonal influenza virus infections and this means hospitals must ensure routine use of influenza molecular assays with high sensitivity and specificity as recommended by NICE, CDC and IDSA.

Consideration should be given to revising current NICE and PHE guidelines for hospitalised patients diagnosed with seasonal influenza virus infection that oseltamivir should be started within 48 hours of onset of influenza symptoms, to align them with CDC and IDSA guidelines, which recommend that oseltamivir treatment for confirmed and suspected hospitalised influenza cases should be started on oseltamivir treatment 75 mg twice daily for 5 days regardless of the duration of influenza illness before hospitalisation .

As new antivirals for influenza viruses are developed, further studies will be required to determine their effectiveness in high-risk patients and inpatient settings.
